# Postpartum follow up of gestational diabetes in a Tertiary Care Center

**DOI:** 10.1186/s13098-017-0303-4

**Published:** 2018-01-03

**Authors:** C. A. Cabizuca, P. S. Rocha, J. V. Marques, T. F. L. R. Costa, A. S. N. Santos, A. L. Schröder, C. A. G. Mello, H. D. Sousa, E. S. G. Silva, F. O. Braga, R. C. Abi-Abib, M. B. Gomes

**Affiliations:** 1 Diabetes Unit, State University of Rio de Janeiro, Rio de Janeiro, Brazil; 2 Rua Cinco de Julho 63/504 Copacabana, Rio de Janeiro, CEP 22051-030 Brazil

**Keywords:** Gestational diabetes, Postpartum glucose testing, Oral glucose tolerance test

## Abstract

**Background:**

Gestational diabetes is a risk factor for future development of type 2 diabetes. The primary aim of this study was to estimate the prevalence of postpartum glucose tolerance status evaluation in pregnancies complicated by gestational diabetes 6–12 weeks after delivery. The secondary one was to identify the factors that are implicated with postpartum glucose retesting.

**Methods:**

This was a retrospective study performed with a cohort of women with gestational diabetes, with prenatal care and delivery at a tertiary care center, from January 2013 to April 2017. The diagnosis of gestational diabetes was based on IADPSG criteria (Fasting ≥ 92 mg/dl, 1 h ≥ 180 mg/dl and/or 2 h ≥ 153 mg/dl, respectively) and the diagnosis of type 2 diabetes and prediabetes were made using the 2016 ADA’s criteria (fasting and 2 h after glucose load ≥ 126 mg/dl and/or ≥ 200 and 100–125 mg/dl and/or 140 and 199 mg/dl, respectively). All women had an appointment scheduled 6–12 weeks postpartum with the results of a 75-g oral glucose tolerance test (OGTT).

**Results:**

Of the 152 evaluated women, 21 (13.8%) returned with the postpartum OGTT results. Of these, 9 (45.0%) had a diagnosis of prediabetes. The use of insulin during gestation was the only factor implicated in a higher adherence rate to postpartum testing OR 6.33 (p 0.002). No significance was found for other demographic and clinical variables (age, family income, years of study, parity, gestational age at first visit, smoking, family history of type 2 diabetes, diagnosis of gestational diabetes before the third trimester, pregestational body mass index, previous history of gestational diabetes and ethnicity).

**Conclusion:**

The majority of patients with gestational diabetes did not return postpartum to perform OGTT and in our study the only factor implicated in a higher postpartum return was the use of insulin during pregnancy. Considering that 45.0% were diagnosed with prediabetes, diabetes care teams should initially identify non-adherent patients.

## Background

Gestational diabetes mellitus (GDM) is a dysglycemia that is first diagnosed in the second or third trimester of pregnancy that is not clearly a preexisting type 1 (T1DM) or type 2 diabetes (T2DM) [1]. It is an important risk factor for permanent or future development of glucose intolerance or T2DM in the postpartum period. About 3–65% of women with previous GDM present T2DM within 5–16 years after pregnancy, depending on the methods used for screening and the studied population [2, 3]. Postpartum glucose tolerance status after GDM varies from 1.1 to 25.3% for T2DM, and from 2.2 to 42.3% for both impaired glucose tolerance and impaired fasting glycemia [4].

Data from Brazilian Institute of Geography and Statistics (IBGE] showed that between 2003 and 2013, obesity among women aged > 20 years rose from 14.0 to 25.2%, with an 11.2% increase [5]. This increase consequently leads to an increase in the cases of GDM [1]. Data from the Brazilian Gestational Diabetes Study Group (EBDG), which enrolled women > 20 years, revealed a prevalence of GDM around 18.0% (based on International Association of the Diabetes and Pregnancy Study Group criteria) [6]. There are well-documented risk factors for GDM such as advanced maternal age, family history of diabetes, previous history of GDM, macrosomia, personal history of polycystic ovary syndrome, non-Caucasian ethnicity, overweight or obesity before pregnancy and multiple gestation [7]. In addition to these risk factors, accumulating data indicate non-classic risk factors for GDM such as low vitamin D and free T4 serum levels, male sex fetuses, in vitro fertilization, shorter legs’ length and epigenetic changes [8–13].

As above mentioned, the higher prevalence of obesity among women of childbearing age is probably increasing the number of pregnant women with undiagnosed T2DM [14], reinforcing data from International Diabetes Federation (IDF) showing that one in two adults with diabetes are unaware about its diagnosis [15]. Consequently, it is recommended to test every women for T2DM at the first prenatal visit. Pregnant women with a fasting glycemia lower than 92 mg/dl in this first visit should perform a 75 g OGTT at 24–28 weeks [1].

In the postpartum diagnostic investigation, fasting glucose compared to the OGTT presented greater reproducibility, but lack of sensitivity [16]. Thus, most postpartum diabetes screening recommendations include the performance of an OGTT [17–19]. Some characteristics observed before, during and after pregnancy can be considered as predictive risk factors for the development of T2DM in the future, such as earlier gestational age at diagnosis of GDM, prepregnancy BMI > 27 kg/m^2^, use of insulin during pregnancy and family history of T2DM in first degree relatives [20, 21]. This postpartum evaluation can identify women who could benefit from preventive intervention measures [17]. However, studies show that even with proper guidelines, the postpartum rates of reevaluation of these patients are low, ranging from 18.5 to 61.0% [18].

Some predictors of the likelihood of postpartum diabetes screening have been described such as older age, ethnicity, insulin use during pregnancy, GDM in a previous pregnancy, higher educational levels, nulliparity, and higher income [16, 22–30]. Prospective studies are warranted to determine how to improve adherence to postpartum screening.

Considering the few data available obtained with the Brazilian population, we carried this study aiming to estimate the prevalence of postpartum glucose tolerance status evaluation in pregnancies complicated by GDM and also to identify the factors that are implicated with postpartum glucose retesting.

## Methods

This was a retrospective cohort study conducted at the Diabetes Unit from Rio de Janeiro's State University (UERJ) from January 2013 until April 2017.

The diagnosis of GDM was based on IADPSG criteria [31]: (Fasting ≥ 92 mg/dl, 1 h ≥ 180 mg/dl and 2 h ≥ 153 mg/dl, respectively. One altered result makes the diagnosis of GDM]. The diagnosis of T2DM and prediabetes at 6–12 weeks postpartum were made using the 2016 ADA’s criteria [3], either through fasting glucose or after the 2 h value of the OGTT. Values equal or greater than 126 and 200 mg/dl in OGTT, respectively, confirmed the diagnosis of T2DM, and between 100–125 mg/dl in fasting and 140 mg/dl and 199 mg/dl, glucose intolerance.

All pregnant women with GDM underwent clinical evaluation and answered a questionnaire with socio-demographic information such as age, self-reported ethnicity (White or non-White), family history of T2DM, prepregnancy BMI (kg/m^2^), time of GDM diagnosis, current pregnancy relevant medical data, marital status, socioeconomic status and income (in US dollars, calculated according to Brazilian Federal Reserve-Banco Central do Brasil—at the time of data collection), years of study, active smoking at initial visit, presence of chronic diseases, regular use of drugs, previous history of GDM, gestational hypertension and a history of a macrosomic newborn. All pregnant women with previous diabetes or using corticoids were excluded.

All patients were instructed to perform a 75-g OGTT between 6 and 12 weeks after delivery and return to an appointment with an endocrinologist in our center. This study was approved by the ethics committee of Pedro Ernesto University Hospital (State University of Rio de Janeiro).

## Statistical analysis

Data are presented as the means (± SD) or medians (minimum–maximum) for continuous variables and numbers (relative frequencies) for discrete variables. To analyze differences between women who returned for postpartum screening and those who did not, we performed the t test or Mann–Whitney test for continuous variables and *x*^*2*^ test or Fisher test for categorical variables, when indicated.

The analysis of the rate of patients returning in the postpartum period with the results of the OGTT, as well as the status of glucose tolerance in this group were also evaluated. Analyses were performed using SPSS version 17.0 (SPSS, Inc., Chicago, Illinois). Multivariate logistic regression was performed with returning patients stratified as follows: (yes, group 1/no, group 0) as dependent variable. Other predictive variables, such as ethnicity, economic status, age, years of school attendance, number of clinical visits during pregnancy, and treatment with insulin (yes/no) were included in the model as independent variables. Odds ratio with 95% CIs were expressed as indicated. A two-sided *p* value less than 0.05 was considered significant.

## Results

### Overview of demographic and clinical data of the studied population

A total of 152 pregnant women were evaluated from January 2013 to April 2017. The clinical and demographic data are shown in Table 1.

**Table 1:** Clinical and demographic data

Variable	Values
Age, years	32 ± 5
Ethnicity, non white n (%)	87 (57.2%)
Family income, U$	646.3 ± 544
Years of study, years	11 ± 2.9
Gestational age at 1st visit, weeks	29 ± 5.7
Diagnosis before 3rd trimester, n (%)	74 (48%)
Smoking, n (%)	18 (11.8%)
Family history of type 2 diabetes, n (%)	74 (48.7%)
Parity, n	1.0 (0–7.0)
Previous GDM, n (%)	17 (13.2%)
Previous BMI, Kg/m^2^	32 ± 7.6
Treatment, n (%)	
Diet	78 (51.3%)
Metformin	10 (6.6%)
Insulin	64 (42.1%)

Data are n (%), mean ± SD or median (min–max)

### Overview of demographic and clinical data of the studied population stratified according to the postpartum return to perform OGTT

Overall, 21 patients (13.8%) returned with the results of the OGTT. A higher frequency of insulin use was noted in the group that performed the OGTT in comparison to those patients who did not (76.2% vs 38.9%, respectively, p value = 0.001). No difference was noted between both groups according to age, family income, years of school attendance, parity, gestational age at first visit, smoking status, family history of T2DM, diagnosis of GDM before the third trimester, pregestational BMI, previous GDM and ethnicity (data shown in Table 2).

**Table 2:** Comparison of clinical and demographic data by adherence to postpartum screening

	Adhrent to postpartum testing	Nonadhrent to postpartum testing	p value
Age, years	32.1 ± 5.6	32.3 ± 5.6	0.866
Ethnicity, non white, n (%)	12 (57.1%)	75 (56.8%)	0.586
Family income, U$	594 ± 319	654 ± 571	0.643
Years of study, years	10.5 ± 2.3	11.1 ± 3.0	0.419
Gestational age at 1st visit, weeks	28.2 ± 5.8	29.2 ± 5.7	0.471
Diagnosis before 3rd trimester, n (%)	11 (52.4%)	64 (49.2%)	0.487
Smoking, n (%)	2 (9.5%)	16 (12.6%)	0.512
Family history of type 2 diabetes, n (%)	10 (47.6%)	64 (56.4%)	0.854
Parity, N	1 (0–3)	1(0–7)	0.708
Previous GDM, n (%)^a^	1 (5.9%)	16 (14.4%)	0.467
Previous BMI, Kg/m^2^	33.97 ± 9.10	31.71 ± 7.32	0.22
Treatment with insulin, yes, n (%)	16 (76.2%)	51 (38.9%)	*0.001*

Data are n (%), mean ± SD or median (min–max)

^a^excluded 24 primiparous women

Among the patients who returned with the OGTT, 9 (45.0%) had the diagnosis of prediabetes. No patient had the diagnosis of T2DM. No difference between these two subgroups was noted regarding age, years of study, parity, gestational age at first visit, smoking, family history of T2DM, diagnosis of GDM before the third trimester, pregestational BMI, previous GDM, ethnicity and insulin use. The family income was higher in the group with abnormal OGTTs (Table 3).

**Table 3:** Comparison of clinical and demographic data by the result of OGTT

	Normal OGTT	Abnormal OGTT	p value
Age, years	32.5 ± 3.7	31.4 ± 7.7	0.659
Ethnicity, non white, n (%)	9 (75%)	3 (33.3%)	0.071
Family income, U$	455 ± 167	779 ± 386	*0.017*
Years of study, years	10.3 ± 2.8	10.8 ± 1.2	0.620
Gestational age at 1st visit, weeks	29.1 ± 5.6	27.0 ± 6.1	0.414
Diagnosis before 3rd trimester, n (%)	6 (50%)	5(55.6%)	0.575
Smoking, n (%)	1 (8.3%)	1 (11.1%)	0.686
Family history of type 2 diabetes, n (%)	6 (54.5%)	4 (44.4%)	0.500
Parity, N	1.5 (0–2)	1(0–2)	0.625
Previous GDM, n (%)	0 (0%)	1 (11.1%)	0.429
Previous BMI, Kg/m^2^	36.4 ± 9.4	30.9 ± 8.1	0.184
Treatment with insulin, yes, n (%)	9 (75%)	7 (77.8%)	0.647

Data are n (%), mean ± SD or median (min–max)

### Multivariate analysis

Multivariate analysis with returning patients to perform OGTT (yes/no) as dependent variable revealed that all the independent variables which entered in the model, even after adjustment, could explain only 14.8% (Nagelkerke R-squared) of a given patient returning to perform the OGTT. The independent variable associated with return in post-partum to perform OGTT was the use of insulin during pregnancy with an odds ratio of 6.333 [B = 1846; 95% CI (1.998–20.076), p = 0.002].

## Discussion

The rate of adhesion to postpartum OGTT found in our study was very low (13. 8%), but similar to that found in other centers where the active search for patients with proactive systems is not part of the routine care [32, 33]. In a study by McCloskey only 23.4% of the women with GDM received any kind of glucose test within 6 months postpartum [32]. Still in agreement with our results, a study conducted in England found that only 18.5% of the women returned for the follow up [33].

The rate of adherence to postpartum screening seems to increase when some type of active search is performed. In a study conducted with 11,825 women with GDM, the rate of fasting glycemia (FPG) or OGTT 6 months postpartum was 50.2%, but 79.1% of these patients measured only FPG and 20.9% performed the OGTT. In this study, containing data collected in different centers, it was not evaluated if the patients received any reminder to perform the OGTT, but it was verified that for women who had postpartum visit the odds of postpartum testing was three times greater than for those who had not [23]. Similar rates of postpartum screening were found in a cohort described by Hunt et al., in which the rate of postpartum screening was 18.0%. After hiring a nurse in order to contact patients during gestation and postpartum for a minimum of three times and providing an OGTT at home, when necessary, this rate increased to 41.0% [25]. In another study, changes in the organization of the health system, initially with the implementation of a program to coordinate the care of patients with GDM by nurses and later with the use of an electronic system to send telephone reminders, the rate of adherence to screening increased from 9.0 to 59.5%, with the first measure, and from 59.5 to 71.5%, with subsequent intervention [34, 35]. In addition, in a randomized controlled trial conducted in Canada, 256 patients with GDM were randomly assigned to four different groups: postal reminders for postpartum OGTT were sent to patients only, for physicians only, for both or none of them. It was shown that in all groups in which reminders were used the rate of adherence to screening was significantly higher than when no reminder was used, reaching 60.5% in the group where reminders were sent to doctors and patients and only 14.3% in the group with no reminder [36]. On the other hand, the same group demonstrated that when trying to implement this measure in practice, although the benefit continued to exist, the effectiveness was not as high as in the controlled study: the adhesion rate in services that did not use the reminders was 13.5% compared to 28.0% for those who sent postal reminders [37]. In our study no type of reminder was sent to patients, which may have contributed to the low adhesion rate.

Even when specific strategies are used to increase adherence to postpartum screening, rates remain unsatisfactory. In studies with questionnaire applications, some of the reasons why patients did not perform the OGTT are lack of time, difficulties with child care, concern only about the risks of GDM to the fetus during pregnancy and less risk awareness to develop diabetes in the future, transport difficulties, among others [38, 39]. Failures in the request of the OGTT by the health care team have been also reported [38]. The factors influencing adherence differ between studies [16, 22, 23, 25–30]. According to a systematic review published by Tovar et al., the main factors associated with higher rates of postpartum screening are higher age, nulliparity, higher income, higher educational level and having been treated with insulin during pregnancy [24]. An association between obesity and a lower chance of adherence has been reported [25, 26], but not confirmed in other studies [16]. In our study, the only factor that was associated with a greater chance of adherence to the OGTT was the use of insulin during gestation. It is possible that the need of insulin therapy may lead to greater awareness of the disease. Several other studies have found similar results to ours in relation to insulin use and increased adherence to screening [16, 26–30]; in other studies this relationship was neutral [22, 23] or inverse [25]. However, as we have found an odds ratio with a higher amplitude, our results must be evaluated with caution. We did not find a relationship between income, family history of diabetes, years of school attendance, ethnicity, BMI, age or previous history of GDM and the chance of adherence to screening. Probably, our sample size could be a limitation to the investigation of these factors. A study conducted also in Rio de Janeiro found that only a previous history of GDM was significantly higher in mothers who returned 6 weeks later for performing the OGTT [2].

Among the patients who underwent postpartum screening, 45% had prediabetes, a rate that may be considered high and confirms the importance of follow-up of these patients. In the Diabetes Prevention Program, a follow up of 10 years, showed that changes in lifestyle and the use of metformin were both effective in reducing the risk of developing diabetes in patients with prediabetes and a previous history of GDM. Compared with placebo, lifestyle changes and metformin reduced the risk of developing diabetes by 35.0 and 40.0%, respectively [40]. Thus, postpartum OGTT is important not only to detect and treat diabetes early when it is already present, but also to target more intensive measures of diabetes prevention in the group that already presents an abnormal OGTT.

## Conclusion

Screening for postpartum diabetes in patients with GDM is a challenge worldwide. In our study, performed in a tertiary care center, a low adherence to diabetes screening after a gestation complicated by GDM was observed and the only factor implicated with returning for diabetes screening was the use of insulin during pregnancy. Active patient search measures need to be implemented in the routine care in a systematic way. Studies evaluating the accuracy of simpler screening tests, such as fasting glucose or the achievement of earlier OGTT in the postpartum period, have already been performed, but they need to be expanded in order to evaluate the practical effectiveness of its use in the follow-up of these patients.
